# MarShip-DET: A Frequency-Aware Multi-Scale Fusion Algorithm for Ship Detection in Maritime Remote Sensing Imagery

**DOI:** 10.3390/s26165295

**Published:** 2026-08-21

**Authors:** Keren Chen, Xufang Zhu, Zhikun Liu, Fuyan Zhao, Kang Wang

**Affiliations:** 1 School of Electronic Engineering, Naval University of Engineering, Wuhan 430033, China; m23285405@nue.edu.cn (K.C.); 0909041023@nue.edu.cn (X.Z.); m25385401@nue.edu.cn (F.Z.); d24382607@nue.edu.cn (K.W.); 2 Air Force Early Warning Academy, Wuhan 430014, China

**Keywords:** ship detection, maritime remote sensing, YOLO11, multi-scale feature fusion, wavelet transform, attention mechanism, CDPFEM, EARCFusion, WavePAM

## Abstract

To address the challenges of multi-scale target variation, complex background interference, and insufficient feature fusion quality in maritime remote sensing ship detection, this paper proposes MarShip-DET, a frequency-aware multi-scale fusion detection algorithm based on YOLO11n. Three core modules are introduced: Channel-Decoupled Progressive Feature Extraction Module (CDPFEM), which employs asymmetric channel decoupling with dual-statistic channel attention and image-relative-position-encoded multi-head self-attention to enhance discriminative feature extraction; Edge-Aware Region Context Fusion Module (EARCFusion), which integrates learnable Sobel edge sensing and cross-attention correction to achieve precise foreground refinement; and Wavelet-guided Prototype Attention Module (WavePAM), which combines Haar wavelet frequency decomposition with prototype-guided spatial compression attention to strengthen deep semantic representation. Experiments on HRSC2016 demonstrate that MarShip-DET achieves an mAP50 of 94.9% and an mAP50-95 of 84.4%, improving by 3.7% and 4.3% over the baseline, respectively. Zero-shot experiments on HRSID and SSDD, including comparisons with YOLO11n, D-FINE-N, and YOLOv13n, provide additional evidence of cross-domain transferability under the evaluated protocol.

## 1. Introduction

With the sustained expansion of the global maritime economy and the growing density of oceanic shipping traffic, maritime security surveillance has emerged as a core strategic imperative for coastal states seeking to safeguard territorial rights and maintain navigational order. Satellite remote sensing, with its capacity for wide-area, all-weather, near-real-time Earth observation, has become an indispensable technological means for perceiving maritime surface targets and grasping situational awareness [1]. Ship detection in maritime remote sensing imagery, as the central task of intelligent maritime monitoring systems, furnishes critical informational support for fisheries management, maritime search and rescue, vessel traffic services, and anti-smuggling operations, holding significant strategic value across both civilian and military domains [2]. Nevertheless, ship targets in remote sensing imagery exhibit substantial scale variation and highly complex backgrounds, often presenting strong directionality and dense clustering; achieving high-accuracy, robust automatic detection against complex maritime backgrounds therefore remains a pressing and insufficiently resolved research challenge in this field.

Prior to the rise of deep learning, maritime remote sensing ship detection relied principally on hand-crafted feature engineering, typically employing descriptors such as HOG and LBP to capture target shape and texture information, and coupling these with classifiers such as SVM or AdaBoost to perform target recognition; some methods further incorporated saliency detection or multi-scale pyramid structures to improve candidate-region recall quality [3]. Such approaches, however, depend heavily on expert knowledge and exhibit weak generalization in the face of complex sea-clutter and multi-scale target scenarios, struggling to reconcile detection speed with accuracy, which limits their application in large-scale operational remote sensing surveillance. Zhao et al. [4] proposed E2YOLOX-VFL on the HRSC2016 dataset [5], introducing efficient channel attention, an improved localization loss, and an equilibrium Gaussian non-maximum suppression strategy, pushing mAP improvement over the baseline by 9.28%. Chen et al. [6] proposed the MSSDet multi-scale ship detection framework and constructed the HRSC2016-MS extended dataset; the joint recursive feature pyramid yielded competitive detection accuracy across multiple datasets. Zhao et al. [2] systematically surveyed the challenges and advances in optical remote sensing ship detection, organizing the improvement strategies and representative schemes for deep learning frameworks under complex maritime scenarios, providing an important reference framework for subsequent research.

Beyond these foundational studies, further works have explored YOLO- and Transformer-based architectures for ship detection, including lightweight variants [7,8,9], diffusion-based enhancement [10], Transformer-based detectors [11,12], multi-scale/rotated designs [13,14], and PVT-enhanced RetinaNet [15], collectively reflecting current diverse architectural trends.

Recent studies further highlight the importance of robust feature representation and rigorous evaluation in remote sensing. Sultan et al. [16] introduced DSCH-Net for physics-inspired and direction-aware remote-sensing image dehazing, illustrating the value of structured representation learning under complex imaging degradation. Zhang et al. [17] developed an R-CNN-based framework for high-resolution remote-sensing ship detection, demonstrating the importance of multi-scale feature representation for ships with substantial scale variation and dense spatial distributions. These studies further motivate reliable and interpretable feature modeling for ship detection under heterogeneous sensor characteristics and complex maritime backgrounds.

Despite the significant progress achieved by these studies, existing methods still face three deep-seated challenges in practical application scenarios. First, backbone networks apply convolution of uniform intensity to all channels, overlooking the intrinsic differences among channels in semantic expressiveness, leading to redundant computational resource allocation; fixed receptive fields, meanwhile, fail to establish global cross-position correlations among ship targets, with missed and false detections particularly prominent in dense occlusion scenarios. Second, cross-layer fusion in the neck typically employs simple concatenation or element-wise addition, lacking explicit alignment capacity for the representational discrepancy between deep abstract features and shallow fine-grained detail; the fusion process also fails to effectively discriminate between target regions and background interference, causing ship edge-contour information to be diluted by background noise. Third, existing deep semantic modeling computes global attention solely within the Euclidean similarity space of pixel tokens, lacking explicit perceptual capacity for multi-frequency structural signals—including edge responses associated with target boundaries and smooth approximations dominated by background regions—embedded in feature maps, causing attention weights to produce diffuse responses in semantically ambiguous regions and constraining overall multi-scale ship detection performance.

It should be emphasized that convolution, channel attention, relative-position self-attention, Sobel operators, cross-attention, Haar wavelets, and prototype aggregation are established techniques. The contribution of MarShip-DET is therefore not the invention of these individual operators. Instead, the methodological novelty lies in their task-oriented coupling within YOLO11n: CDPFEM performs asymmetric channel decoupling and switches the global modeling strategy with network depth; EARCFusion converts learnable edge responses into foreground and confusion-region context vectors that explicitly condition cross-attention; and WavePAM couples wavelet-derived frequency guidance with prototype-based spatial compression in the deepest semantic stage. The study evaluates whether these three coordinated design choices are complementary for multi-scale ship detection rather than treating the constituent primitives as independently novel.

Although the three modules are designed to address scale variation, cluttered backgrounds, and insufficient multi-frequency representation, the correspondence between a module and a target challenge constitutes a design rationale rather than empirical evidence of effectiveness. Accordingly, repeated-seed ablation, scale-stratified evaluation, scenario-oriented diagnostics, and unified baseline comparisons are used to examine whether the proposed mechanisms provide consistent improvements under the evaluated conditions.

To address the above limitations, the proposed MarShip-DET adopts a frequency-aware adaptive fusion strategy for ship detection in maritime remote sensing imagery, built upon YOLO11n with systematic improvements across backbone feature extraction, deep semantic modeling, and neck cross-layer fusion. The principal contributions are as follows:

1. CDPFEM is proposed. Following the partial convolution philosophy, the channel dimension is asymmetrically decoupled into a local convolution subset and a global attention subset. In shallow layers, Dual-Statistic Channel Attention (DSCA) is introduced to achieve adaptive channel re-weighting via mean and standard deviation dual statistics; in deep layers, an image-relative-position-encoded multi-head self-attention mechanism is integrated to explicitly model the spatial topology among pixels. Under the evaluated configuration, this design improves discriminative feature extraction for multi-scale ship targets while maintaining a controlled computational cost.
2. EARCFusion is proposed. A Cross-Sample Memory-Guided Attention Unit (CSMGAU) is introduced to enhance global context perception; thereafter, learnable Sobel filters dynamically partition the spatial domain into foreground confidence regions and confusion regions, and region-aware context vectors guide a cross-attention mechanism to perform fine-grained correction of target features. This design improves semantic alignment and region discrimination during cross-layer fusion.
3. WavePAM is proposed. Haar wavelet transform is applied for multi-subband frequency decomposition of deep features; Local-Global Dual-path Channel Weighting Unit (LGDCWU) is introduced for dynamic balanced fusion of high-frequency edge responses and low-frequency semantic constraints; and a spatial compression attention branch based on a learnable prototype matrix is constructed for adaptive aggregation of global features. The resulting dual-path formulation combines frequency-aware guidance and prototype-based spatial aggregation for deep semantic representation.

## 2. Related Work

### 2.1. YOLO11 Baseline

YOLO11 [18] is a next-generation real-time object detection framework released by Ultralytics in 2024, which introduces several key improvements while continuing the YOLO series’ unified pipeline for rapid single-pass inference. The backbone replaces the conventional C2f bottleneck block with an improved C3k2 module, efficiently extracting multi-level semantic features via cross-stage feature aggregation; an SPPF module extends the multi-scale receptive field through cascaded max-pooling; and a C2PSA module introduced at the tail of the backbone performs global context modeling of the deepest features via multi-head self-attention, enhancing semantic understanding of targets in complex scenes. The neck employs bidirectional feature pyramids for hierarchical cross-scale representation aggregation, and the decoupled prediction branch independently regresses bounding boxes and classifies targets at P3, P4, and P5 scales. YOLO11 attains a competitive trade-off across detection accuracy, computational efficiency, and model compactness, demonstrating strong performance on multiple standard detection benchmarks, and provides a solid foundational architecture for the targeted improvements toward maritime remote sensing scenarios proposed in this paper.

### 2.2. Related Work on Feature Fusion Modules

Multi-scale feature fusion is the central design problem of detector neck networks. FPN achieves cross-scale fusion via top-down progressive semantic propagation and has been widely adopted as the standard neck paradigm; PANet introduces an additional bottom-up path to strengthen the propagation of shallow spatial detail; BiFPN employs a bidirectional weighted fusion strategy for efficient multi-scale information aggregation. Addressing the specific demands of remote sensing image object detection, researchers have proposed more targeted fusion schemes: Zhang et al. [19] proposed GFNet, a global fusion network that maximizes feature information utilization via a large-backbone–small-neck strategy and achieves lossless fusion of non-adjacent layers; Qu et al. [20] designed a feature fusion and distribution module under the YOLOXs framework, using an attention mechanism to compensate for the information loss of FPN in non-adjacent-layer fusion; Meng et al. [21] proposed C-AFBiFPN for SAR ship detection, integrating convolutional feature enhancement and BiFormer attention within a BiFPN strategy to improve cross-scale global modeling capability; Duan et al. [22] proposed TA-FPN, a dual-layer attention feature pyramid network that introduces a small-target detail enhancement module to improve feature expressiveness for dense targets. These studies demonstrate that introducing attention guidance, region awareness, and edge enhancement into feature fusion yields substantial benefits for improving multi-scale fusion quality under complex backgrounds, providing important references for the design of the proposed EARCFusion module.

## 3. Method

**Figure 1:**
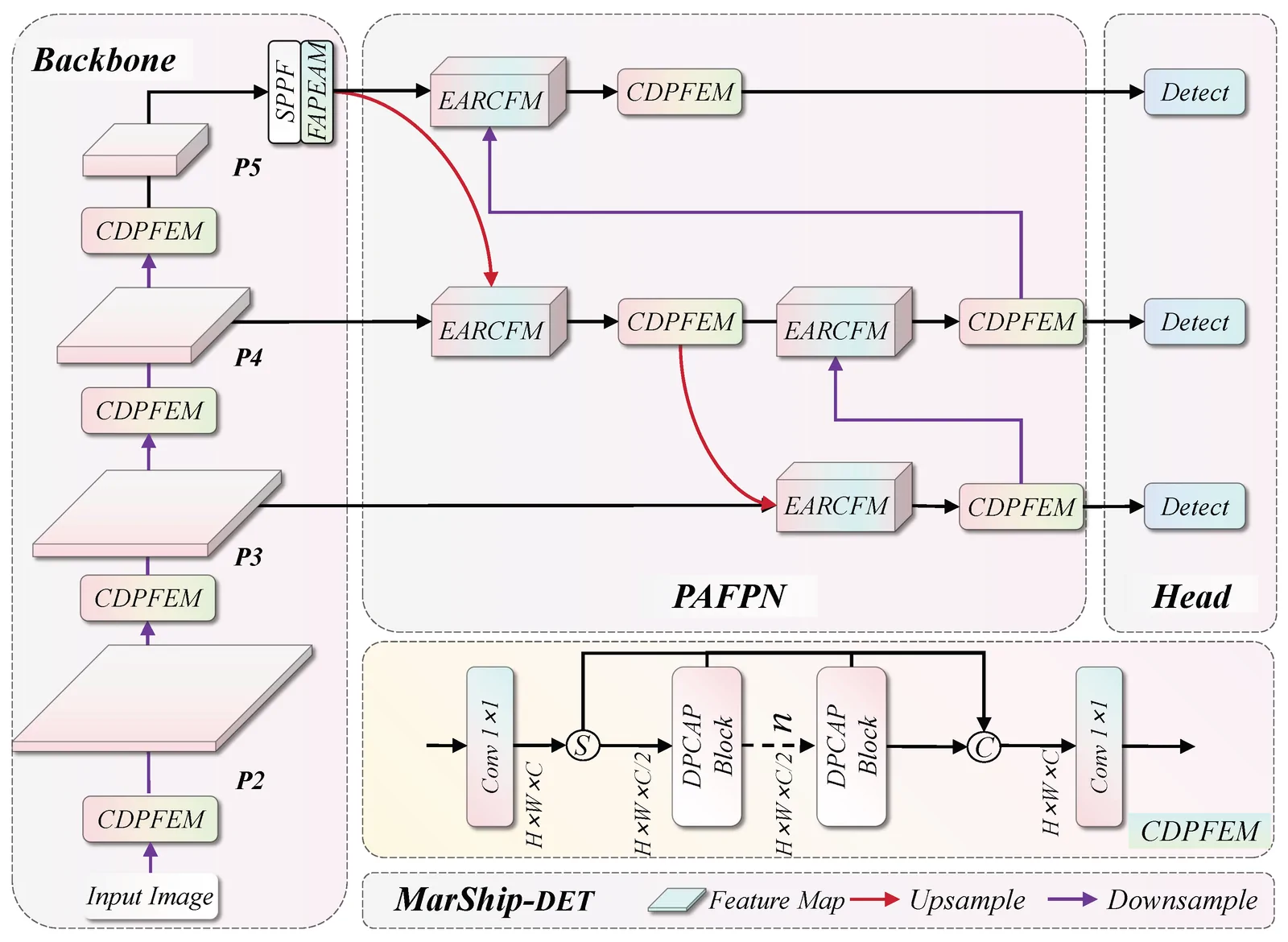
Overall network architecture of MarShip-DET.

This paper takes YOLO11n as the foundational architecture and performs systematic improvements along three dimensions—backbone feature extraction, deep semantic modeling, and neck cross-layer fusion—to address the detection challenges posed by substantial scale variation and complex background interference of ship targets in maritime remote sensing imagery. The improved overall architecture is shown in Figure 1. The input remote sensing image undergoes initial downsampling before entering the multi-level feature extraction backbone constructed from CDPFEM: shallow CDPFEM efficiently extracts local ship texture features via channel-decoupled partial convolution and DSCA, while deep CDPFEM further establishes long-range cross-spatial dependencies via an image-relative-position-encoded multi-head self-attention mechanism, generating backbone features at the P3, P4, and P5 scales in succession. Following multi-receptive-field aggregation by an SPPF module at the backbone tail, the features are fed into WavePAM for wavelet frequency decomposition and dual-path prototype-enhanced attention modeling, producing a deep global feature representation that combines high-frequency edge responses with low-frequency semantic constraints. In the neck fusion stage, all cross-layer fusion nodes within the bidirectional feature pyramid are replaced with EARCFusion, which enhances global context via CSMGAU and then decomposes the spatial domain into foreground and confusion regions via Sobel edge perception, guiding a cross-attention mechanism to perform fine-grained correction of the fused features. The multi-scale features, after further refinement by C3k2, are passed to the detection head, which performs target classification and bounding box regression at the P3, P4, and P5 outputs, completing multi-scale precise detection of ship targets on the maritime surface.

### 3.1. CDPFEM Feature Extraction Module

**Figure 2:**
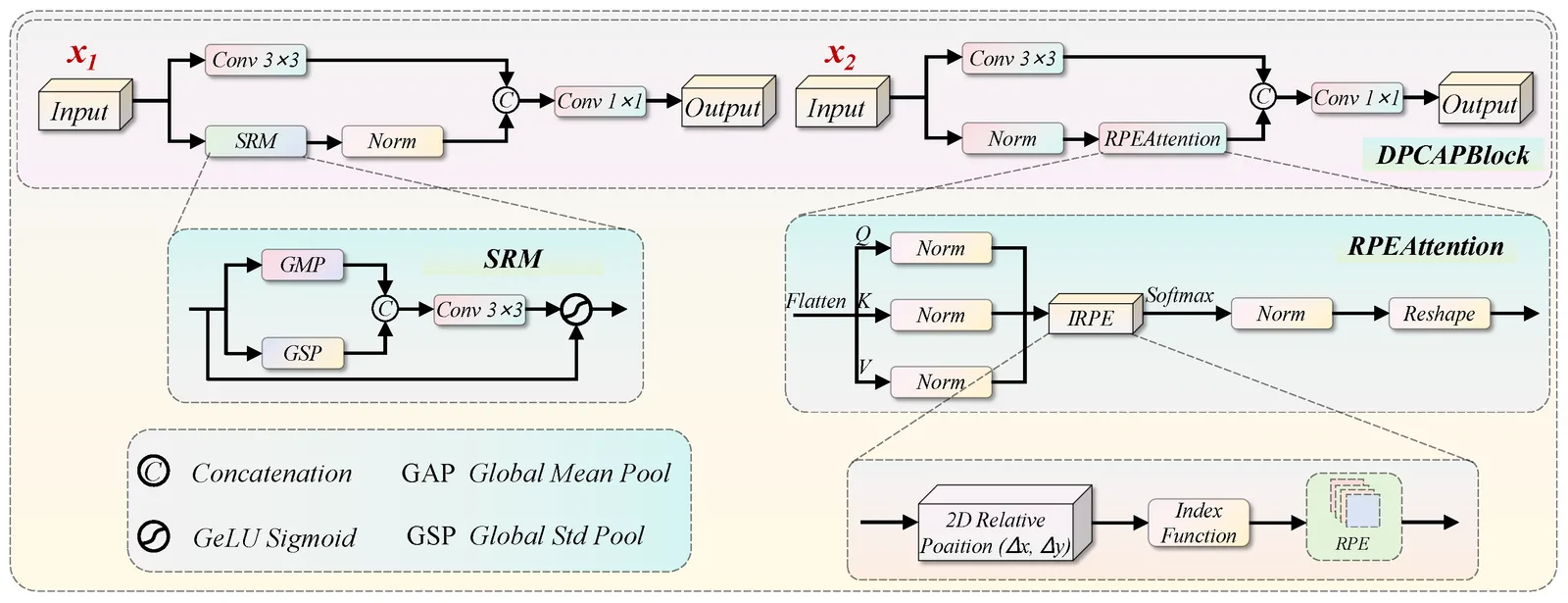
Structure of the CDPFEM module.

When performing feature extraction, the conventional C3k2 module applies convolution of uniform intensity to all channels, overlooking the intrinsic differences among channels in semantic expressiveness, resulting in redundant computational resource allocation and imbalanced feature expressiveness. Moreover, standard convolution kernels are constrained by a fixed receptive field and cannot establish long-range pixel dependencies; in maritime remote sensing imagery, ship targets exhibit considerable scale variation and high visual similarity to complex maritime backgrounds, imposing heightened demands. To address these issues, this paper proposes CDPFEM: the module takes the partial convolution philosophy as its core, decoupling the channel dimension into a local convolution processing subset and a global attention processing subset; in shallow stages it introduces DSCA, and in deep stages it introduces a multi-head attention mechanism carrying image-relative-position encoding, achieving cooperative optimization of computational efficiency and feature expressiveness, and effectively mitigating missed and false detections under dense targets, small targets, and strongly interfering backgrounds. The module structure is shown in Figure 2.

CDPFEM modifies the cross-stage feature aggregation framework of the classic C3k2 by replacing the standard bottleneck block with a Dual-path Partial Convolution Adaptive Perception Block (DPCAPBlock), constructing a feature extraction backbone with channel-decoupled perception capability. Specifically, given an input feature map $X\in R^{B\times C_{1}\times H\times W}$, the module first expands the channel count to 2c via a 1×1 convolution $W_{1}$ (where $c=\lfloor C_{2}\cdot e\rfloor$ and *e* is the expansion ratio), then equally splits along the channel dimension into two initial feature streams $f_{0}$ and $f_{1}$; $f_{1}$ is fed sequentially into *n* cascaded DPCAPBlocks for incremental feature refinement, with each level’s output recorded as $f_{2},\ldots ,f_{n}$; finally all intermediate features are concatenated along the channel dimension and merged by a 1×1 convolution $W_{2}$. The above process is uniformly expressed as

(1) $$Y=W_{2}\cdot \mathrm{Cat}\left(f_{0},f_{1},f_{2},\ldots ,f_{n}\right),\;f_{i}=B_{i}\left(f_{i-1}\right),\;\left[f_{0},f_{1}\right]=\mathrm{Split}\left(W_{1}\cdot X\right),\;i=1,2,\ldots ,n$$

where $B_{i}\left(\cdot \right)$ denotes the *i*-th DPCAPBlock submodule and Cat(·) denotes concatenation along the channel axis. This multi-path progressive feature aggregation mechanism grants the module gradient accumulation capability for target features at different abstraction levels, enabling the final output feature map to simultaneously carry shallow texture and deep semantic information, effectively improving the representational richness for multi-scale ship targets.

The core design idea of DPCAPBlock is to asymmetrically decouple the channel space of the input features: a small subset of channels undergoes locally precise convolutional transformation, while the remaining channels undergo lightweight or enhanced attention perception, thereby obtaining richer feature representations with a modest parameter increase. Given an input $x\in R^{B\times C\times H\times W}$, the module uses $n_{\mathrm{div}}=4$ as the partitioning coefficient, assigning the first $\left\lfloor C/n_{\mathrm{div}}\right\rfloor$ channels as the active component $x_{1}$ for convolutional computation and the remaining channels as the global component $x_{2}$ for attention modeling; $x_{1}$ is immediately processed by a 3×3 partial convolution to capture local geometric texture features:

(2) $$\left[x_{1},x_{2}\right]=\mathrm{Split}\;\left(x,\;\left\lfloor \frac{C}{n_{\mathrm{div}}}\right\rfloor ,\;C-\left\lfloor \frac{C}{n_{\mathrm{div}}}\right\rfloor \right)$$

This asymmetric partitioning strategy ensures that convolutional parameters are concentrated on the channel subset of higher information density, thereby significantly reducing invalid computational overhead. For the global component $x_{2}$, DPCAPBlock dynamically selects the attention strategy according to the hierarchical depth of the network. In shallow stages, DSCA extracts the per-channel mean $\mu_{x_{2}}$ and standard deviation $\sigma_{x_{2}}$ as style descriptors; after cross-statistic convolutional mapping, channel gating weights are generated to perform adaptive feature re-weighting on $x_{2}$

(3) $$g=\sigma_{h}\;\left(\mathrm{BN}\;\left({\mathrm{Conv}}_{1\times 2}\;\left([\mu_{x_{2}};\;\sigma_{x_{2}}]\right)\right)\right),\;x_{2}^{\prime }=x_{2}⊙g$$

where $\mu_{x_{2}},\sigma_{x_{2}}\in R^{B\times C_{2}\times 1\times 1}$ are the global mean and standard deviation along the channel dimension, respectively; $\sigma_{h}\left(\cdot \right)$ is the HardSigmoid activation; ⊙ denotes element-wise multiplication; and BN(·) is batch normalization. In deep stages, a multi-head self-attention mechanism integrated with image two-dimensional relative position encoding is employed; by introducing a Euclidean distance positional bias term $B_{\mathrm{RPE}}\in R^{n_{h}\times N\times N}$ (quantized via a piecewise index function) into the key-vector attention matrix, the spatial topology among feature pixels is explicitly modeled, enhancing the model’s awareness of target morphology and relative positions

(4) $$\mathrm{Attn}\left(Q,K,V\right)=\mathrm{Softmax}\;\left(\frac{QK^{⊤}+B_{\mathrm{RPE}}}{\sqrt{d_{k}}}\right)V,\;x_{2}^{\prime }=\mathrm{Attn}\left(\mathrm{Proj}\left(\mathrm{LN}\left(x_{2}\right)\right)\right)$$

where $Q,K,V\in R^{B\cdot n_{h}\times N\times d_{k}}$ are the query, key, and value matrices after linear projection; N=H×W is the spatial sequence length; $d_{k}$ is the per-head dimension; LN(·) is two-dimensional layer normalization; and the encoding index of $B_{\mathrm{RPE}}$ is determined by a piecewise linear-logarithmic composite function, effectively compressing the representation space for long-range positional relationships. CDPFEM introduces differentiated channel decoupling and attention mechanisms at different depth levels of the backbone network, significantly enhancing the network’s multi-scale feature extraction and discrimination capability for ship targets in maritime remote sensing imagery while keeping the overall parameter scale controllable. The lightweight channel attention in shallow layers enables the network to obtain enhanced responses to ship texture and edge features at low semantic levels, while the image-relative-position-encoded multi-head attention mechanism in deep layers effectively establishes long-range dependencies across spatial positions, improving recognition capability for densely docked and occluded targets.

### 3.2. EARCFusion Feature Fusion Module

**Figure 3:**
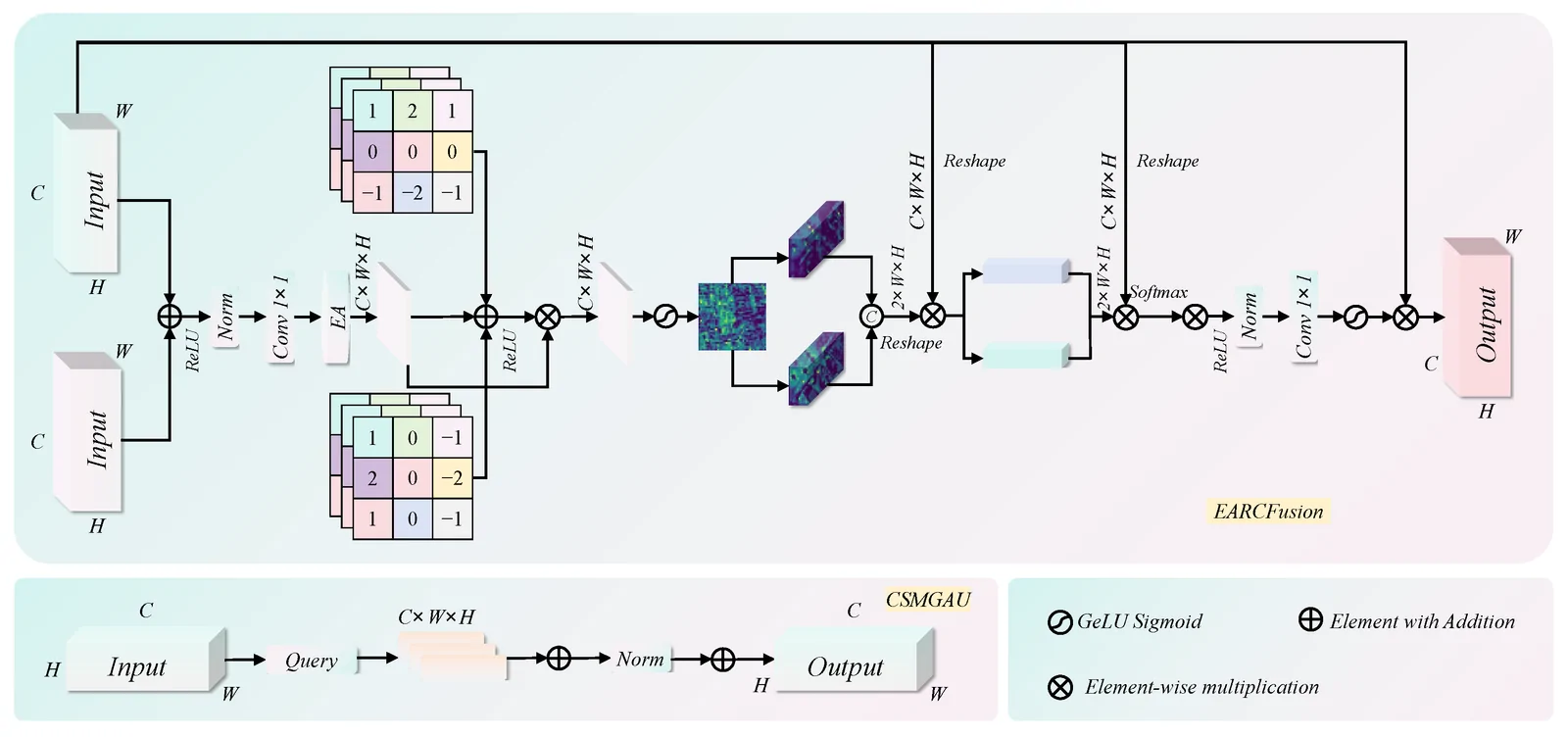
Structure and information flow of the EARCFusion module.

When performing multi-scale feature fusion, traditional feature pyramid networks typically aggregate features from different hierarchical levels via simple upsampling-concatenation or element-wise addition. Such operations lack explicit alignment capacity for cross-layer semantic discrepancies, giving rise to a semantic gap between high-level semantic information and low-level spatial detail, and the resulting fused feature representations are of limited quality. Concurrently, the fusion process lacks an effective mechanism to distinguish target regions from background interference, making it difficult for the network to focus on ship target edge contours and local texture details when confronted with complex maritime backgrounds, causing feature response blur and dilution. To address these issues, this paper proposes EARCFusion: on the basis of cross-layer feature alignment, the module introduces CSMGAU to enhance global feature expression; it then applies Sobel operators to the fused features for edge perception, dynamically partitioning the spatial domain into foreground and confusion regions; these serve as guidance for constructing region-aware context vectors; and finally a cross-attention mechanism performs fine-grained correction of target features, achieving coordinated fusion of semantic alignment, edge perception, and attention guidance, and effectively improving ship target detection accuracy against complex maritime backgrounds. The module structure is shown in Figure 3.

As illustrated in Figure 3, the two adjacent-scale inputs are first channel-aligned and preliminarily fused. CSMGAU then injects global memory-guided context. A learnable Sobel branch converts the enhanced feature into foreground-confidence and confusion-region maps, which are compressed into region-aware context vectors. These vectors provide keys and values for cross-attention, while the original feature provides queries; the resulting attention response gates the target feature to produce the refined fusion output.

EARCFusion receives feature maps g and x from two adjacent feature levels as input, first performs channel alignment on each via 1×1 convolution to obtain $\bar{g}=W_{g}\left(g\right)$ and $\bar{x}=W_{x}\left(x\right)$, then applies element-wise addition to the aligned features and feeds the result into a 3×3 convolutional layer for preliminary semantic fusion. The fused result is further enhanced by CSMGAU for global context, yielding the enhanced fusion feature F. CSMGAU employs learnable memory units $M_{k}\in R^{S\times C}$ and $M_{v}\in R^{S\times C}$ to implicitly model cross-sample correlations across all samples, replacing the costly $O(N^{2})$ computation of self-attention. The computation is expressed as

(5) $$F=\mathrm{ReLU}\;\left(\tilde{F}+M_{v}^{⊤}\cdot \mathrm{Norm}\;\left(\mathrm{Softmax}\;\left({\tilde{F}}_{r}\cdot M_{k}^{⊤}\right)\right)\right),\;\tilde{F}={\mathrm{Conv}}_{3\times 3}\left(\bar{g}+\bar{x}\right)$$

where ${\tilde{F}}_{r}\in R^{B\times HW\times C}$ is $\tilde{F}$ flattened into a spatial sequence, Norm(·) denotes $l_{1}$ normalization along the memory dimension, and *S* is the number of memory slots. This mechanism requires only O(N·S) computational complexity to effectively aggregate global context information, significantly enhancing the semantic responsiveness of the fused features to target content while maintaining a lightweight design.

After obtaining the enhanced fusion feature F, EARCFusion enters the edge perception and region decomposition stage. The module applies a learnable Sobel filter to F to extract the edge response map S(F), which is then mapped via 1×1 convolution and Sigmoid activation to a spatial probability map, followed by a piecewise transformation that dynamically partitions spatial pixels into foreground and confusion-region categories

(6) $$p=\sigma \;\left({\mathrm{Conv}}_{1\times 1}\;\left(S(F)\right)\right)-0.5,\;f_{\mathrm{fg}}=\mathrm{clip}\left(p,\;0,\;1\right),\;f_{\mathrm{cg}}=0.5-\left|p\right|$$

where $f_{\mathrm{fg}}$ and $f_{\mathrm{cg}}$ represent the spatial weight maps for the foreground confidence region and the target–background confusion region, respectively. The concatenated probability map $P=\left[f_{\mathrm{fg}};\;f_{\mathrm{cg}}\right]\in R^{B\times 2\times HW}$ is then matrix-multiplied with the flattened feature $x_{r}\in R^{B\times HW\times C}$, compressing the spatially distributed information into compact region-aware context vectors $C=P\cdot x_{r}\in R^{B\times 2\times C}$. The context vector C is separately transformed by key convolution $\phi_{k}\left(\cdot \right)$ and value convolution $\phi_{v}\left(\cdot \right)$; the original feature x is transformed by query convolution $\phi_{q}\left(\cdot \right)$; together, these form the inputs for the cross-attention computation. The attention weight matrix is normalized with a $C^{-1/2}$ scaling factor applied to the inner product of Q and K; after Softmax normalization, the values are weighted-summed to obtain the refined context A, which is then used to apply channel-wise scale modulation to the original features via Sigmoid gating

(7) $$A=\mathrm{Softmax}\;\left(\frac{QK^{⊤}}{\sqrt{C}}\right)V,\;Y=\mathrm{ReLU}\;\left(x⊙\sigma \;\left({\mathrm{Conv}}_{3\times 3}\left(A\right)\right)\right)$$

where $Q=\phi_{q}\left(x\right)\in R^{B\times HW\times C}$, $K=\phi_{k}\left(C\right)\in R^{B\times C\times 2}$, $V=\phi_{v}\left(C\right)\in R^{B\times 2\times C}$. This design enables the model to use the compressed semantics of foreground and confusion regions as guidance to selectively correct the spatial response intensity of target features, thereby suppressing background noise interference and enhancing discriminative capability at ship boundary regions.

EARCFusion organically integrates CSMGAU enhancement, edge-guided region decomposition, and region-aware cross-attention correction within a unified framework, fundamentally overcoming the inherent limitations of conventional feature fusion strategies in semantic alignment and region discrimination. Deployed at all cross-layer feature fusion nodes of the detection head, the module ensures that features at different scales each receive adaptive enhancement targeted at foreground target regions during fusion, effectively suppressing complex maritime background interference while markedly improving the retention quality of edge detail.

### 3.3. WavePAM Module

**Figure 4:**
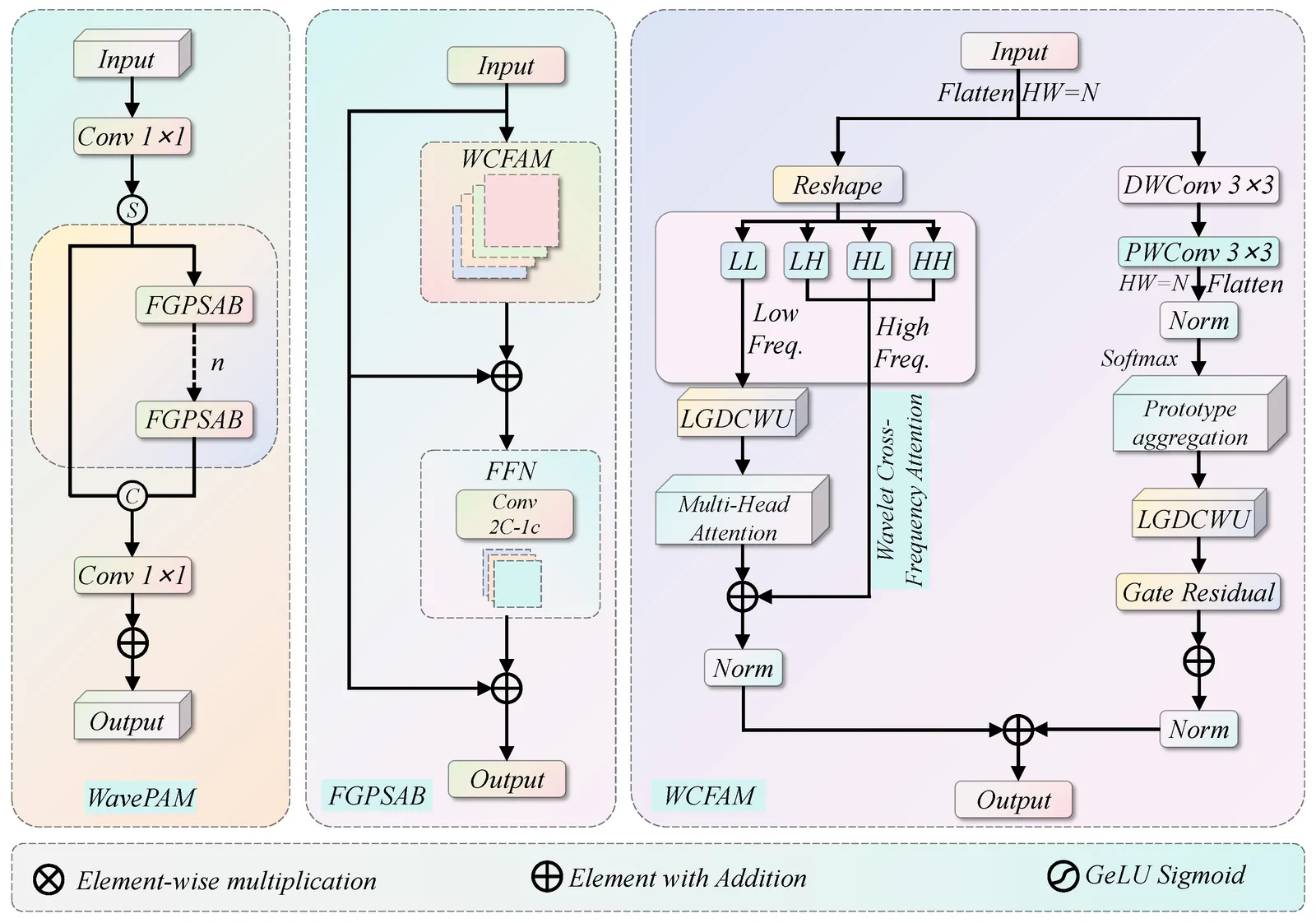
Structure and information flow of the WavePAM module.

The C2PSA module employed at the end of the original YOLO11 backbone relies on standard multi-head self-attention for global context modeling of the deepest features. This mechanism, however, computes similarity solely within the Euclidean space of pixel tokens, lacking explicit perceptual capacity for the multi-frequency signal components embedded in feature maps; it cannot effectively distinguish the high-frequency edge information corresponding to target structure from the low-frequency smooth information dominated by background regions, causing attention weights to produce diffuse responses in semantically ambiguous areas. Meanwhile, the computational cost of standard self-attention grows quadratically with sequence length when processing large-scale deep feature maps, constraining the module’s practical utility. To address these limitations, this paper proposes WavePAM: using wavelet transform as a frequency analysis tool, the module performs multi-subband decomposition and adaptive frequency weighting of features prior to attention computation, and introduces a spatial compression attention branch based on learnable prototypes; from the dual perspectives of frequency perception and prototype guidance, this design cooperatively enhances the semantic representation capacity of deep features, effectively improving the model’s global awareness of and detail retention for ship targets against complex maritime backgrounds. The module structure is shown in Figure 4.

As illustrated in Figure 4, WavePAM first splits the input into a bypass path and a processing path. Within each FGPSABlock, the processing path forms two complementary branches: the frequency branch applies Haar decomposition and LGDCWU-guided cross-attention, whereas the prototype branch compresses spatial tokens with a learnable prototype matrix and performs adaptive channel correction. The two branches are merged and combined with the residual feedforward path before cross-stage aggregation with the bypass feature.

WavePAM adopts the dual-path cross-stage aggregation framework of C2PSA. For an input feature map $X\in R^{B\times C\times H\times W}$, the channel count is first expanded to 2c via a 1×1 convolution $W_{1}$ (where c=⌊C·e⌋), then equally split along the channel dimension into a static bypass a and a processing main path b; b undergoes iterative enhancement through *n* serially connected Frequency-Guided Position-aware Self-Attention Blocks (FGPSABlocks), and the result is concatenated with a before being integrated by a 1×1 convolution $W_{2}$

(8) $$Y=W_{2}\cdot \mathrm{Cat}\;\left(a,\;M(b)\right),\;\left[a,\;b\right]=\mathrm{Split}\left(W_{1}\cdot X\right)$$

where M(·) denotes the serial processing sequence composed of *n* FGPSABlocks. Each FGPSABlock replaces the original multi-head self-attention layer in the standard PSABlock with a Wavelet Cross-Frequency Attention Module (WCFAM), retaining the feedforward network branch and residual connection structure; the residual path ensures gradient flow while enabling effective stacking of attention-enhanced features with feedforward nonlinear transformations.

The core design of WCFAM lies in introducing wavelet frequency decomposition into the attention computation process, enabling the model to perform adaptive multi-scale perception of features in the frequency domain. Given an input feature map $X\in R^{B\times C\times H\times W}$, the module first applies shared Haar wavelet convolution kernels to perform one-level 2-D discrete wavelet decomposition, extracting high-frequency subbands in the horizontal, vertical, and diagonal directions while retaining the overall structural information in the low-frequency approximation subband, yielding four frequency components with halved spatial resolution

(9) $$F_{LL},\;F_{LH},\;F_{HL},\;F_{HH}=W\left(X\right),\;F_{hi}=F_{LH}+F_{HL}+F_{HH},\;F_{l}=F_{LL}$$

where W(·) denotes the fixed-weight Haar wavelet transform operator, whose convolution kernels are composed of the outer products of the low-pass filter $\psi_{l}=\frac{1}{\sqrt{2}}{\left[1,\;1\right]}^{⊤}$ and the high-pass filter $\psi_{h}=\frac{1}{\sqrt{2}}{\left[-1,\;1\right]}^{⊤}$, shared across all channels and not updated during backpropagation. The high-frequency component $F_{hi}$ concentrates the discriminative edge responses corresponding to ship contours, while the low-frequency component $F_{l}$ retains the smooth approximation of the target’s overall structure. To adaptively balance the contributions of these two frequency components in guiding attention, WCFAM introduces LGDCWU, which generates soft attention gating weights via the superposition of a local token-level linear transform and a global mean-pooling-level linear transform, dynamically fusing the high- and low-frequency features:

(10) $$F_{\mathrm{fre}}=\omega ⊙F_{hi}+F_{l},\;\omega =\sigma \;\left(W_{l}\;\left(F_{hi}+F_{l}\right)+W_{g}\;\bar{\left(F_{hi}+F_{l}\right)}\right)$$

where $W_{l},W_{g}\in R^{C\times C}$ are the local and global attention mapping matrices, respectively; $\bar{\left(\cdot \right)}$ denotes mean pooling over the token sequence. The fused frequency feature $F_{\mathrm{fre}}$ serves as keys and values in subsequent multi-head cross-attention computation, guiding query token responses with frequency-aware context vectors; the first-branch output is $x_{1}=\mathrm{LN}\;\left(\mathrm{MHA}(Q,\;F_{\mathrm{fre}},\;F_{\mathrm{fre}})+Q\right)$, where Q is the token sequence obtained by flattening the input features and LN(·) is layer normalization.

In the second branch, WCFAM aggregates spatial tokens into *M* compact prototype vectors via a learnable prototype head matrix $M_{h}\in R^{M\times C}$, using a spatially-normalized weight matrix as aggregation coefficients to achieve adaptive sampling and compression of high-information-density spatial regions:

(11) $$P={\mathrm{Softmax}}_{\mathrm{spatial}}\;\left(\bar{F}\left(X\right)\cdot M_{h}^{⊤}\right)$$

where ${\bar{F}}_{r}\in R^{B\times N\times C}$ is the flattened token sequence of the source features, $\bar{F}\left(\cdot \right)$ denotes the flatten operation following local enhancement via depthwise separable 3×3 convolution, and ${\mathrm{Softmax}}_{\mathrm{spatial}}\left(\cdot \right)$ denotes Softmax normalization along the spatial dimension, enabling each prototype to selectively aggregate information from global spatial positions in an attention-weighted manner. The resulting prototypes P are aligned in sequence length with the query tokens Q and added to them; LGDCWU again generates channel-wise scale gating coefficients α, which adaptively correct the query features in a multiplicative-residual fashion to obtain the second-branch output $x_{2}=\mathrm{LN}\left(Q⊙\alpha +Q\right)$. The two branch outputs are finally added element-wise to produce the complete enhanced representation $x_{\mathrm{out}}=x_{1}+x_{2}$. This dual-path parallel structure makes frequency-domain global context modeling and prototype-driven spatial compression attention complementary, capturing long-range dependencies while preserving sensitivity to the fine local structure of targets.

WavePAM effectively compensates for the deficiencies of standard self-attention in frequency perception and spatial information compression by embedding wavelet frequency decomposition and the dual-path adaptive attention mechanism into the deepest feature aggregation node of the backbone. The wavelet-guided frequency weighting mechanism enables the network to adaptively emphasize high-frequency edge structural responses corresponding to ship targets while retaining low-frequency semantic background constraints; the prototype aggregation branch compresses global features into compact, discriminative representations via learnable spatial sampling weights, reducing background redundancy interference on attention computation. The ablation experiments in Section 4 quantitatively assess the contribution of this design to deep semantic representation and detection performance on HRSC2016.

## 4. Experimental Results and Analysis

### 4.1. Experimental Datasets

HRSC2016 (High-Resolution Ship Collections 2016) [5] serves as a widely adopted high-resolution evaluation benchmark for ship detection and recognition in remote sensing imagery, constructed by Northwestern Polytechnical University in 2016. All images were collected from Google Earth, comprising 1061 labeled images and 2976 ship instances spanning 28 fine-grained ship categories, including aircraft carriers, destroyers, and merchant vessels, with precise oriented bounding box annotations. The dataset simultaneously provides horizontal boxes, oriented boxes, and pixel-wise segmentation masks as three annotation formats, featuring rich multi-scale targets and complex maritime backgrounds, making it among the most representative evaluation benchmarks in remote sensing ship detection. The official split divides the dataset into independent training (436 images), validation (181 images), and test (444 images) partitions, ensuring strict separation and result comparability across model training, tuning, and evaluation.

All HRSC2016 experiments in this study are formulated as horizontal bounding-box detection to remain consistent with the standard YOLO11 detection head. Because HRSC2016 provides horizontal boxes in addition to oriented boxes and segmentation masks, the horizontal annotations are used for training and evaluation, and the reported IoU, mAP50, and mAP50-95 values refer to horizontal boxes. Oriented-box IoU and rotated detection metrics are therefore outside the scope of the present experiments and should not be inferred from the reported results.

To further assess the out-of-domain transferability of the proposed approach, this study incorporates HRSID (High-Resolution SAR Image Dataset) [23] and SSDD (Ship Synthetic Aperture Radar Dataset) [24], two synthetic aperture radar (SAR) image datasets, as held-out generalization test sets. HRSID integrates observational data from Sentinel-1B, TerraSAR-X, and TanDEM across multiple platforms, covering three spatial resolution levels of 0.5 m, 1 m, and 3 m, comprising 5604 SAR patch images of 800 × 800 pixels with a cumulative 16,951 annotated ship targets, characterized by complex multi-scale target distributions and strong background clutter. SSDD originates from RadarSat-2 satellite imagery, containing 1160 images of 500 × 500 pixels with 2456 annotated ship instances at spatial resolutions spanning 1 to 15 m, encompassing varied sea states and imaging modes with considerable variation in target size, pose, and noise level. Both datasets differ substantially from the primary training set HRSC2016 in imaging mechanism, data source, and scene distribution, enabling effective examination of model robustness and generalization capability away from the training domain from different angles.

### 4.2. Experimental Setup and Parameter Configuration

All experiments in this paper were conducted under a unified, fixed hardware and software environment to ensure reproducibility and fair comparative evaluation. The hardware configuration is as follows: the processor is an Intel Core i9-13900K with 24 cores at a base clock of 3.0 GHz; the GPU is an NVIDIA GeForce RTX 4090 with 24 GB VRAM; total system memory is 64 GB DDR5. On the software side, the operating system is Ubuntu 22.04 LTS; the deep learning framework is PyTorch 2.1.0 with Python 3.10; parallel acceleration uses CUDA 12.1 and cuDNN 8.9.2; and the entire model was developed, trained, and validated within the Ultralytics YOLO11 open-source framework.

Training hyperparameters are uniformly set as follows: batch size: 16; input image resolution: 640×640; total training epochs: 200. The initial base learning rate is 0.01, with cosine annealing for dynamic decay throughout training; the optimizer is stochastic gradient descent (SGD) with a momentum of 0.937 and a weight decay coefficient of 0.0005; the warm-up duration is 3 epochs. Data augmentation strategies including Mosaic, MixUp, and random affine transformations, along with all other unspecified hyperparameters, follow the original default YOLO11 configuration.

For the core ablation study, each configuration was independently trained three times using random seeds of 0, 42, and 3407. The checkpoint with the highest validation-set mAP50-95 within each run was retained for final testing. Validation uses the same 640×640 input size for all models, with a confidence threshold of 0.001, an NMS IoU threshold of 0.7, and a maximum of 300 detections per image. The augmentation configuration is fixed across all runs: Mosaic probability: 1.0, MixUp probability: 0.0, horizontal-flip probability: 0.5, vertical-flip probability: 0.0, translation: 0.1, scale: 0.5, rotation: 0.0 degrees, shear: 0.0 degrees, and perspective: 0.0. No model-specific augmentation tuning was performed, and the same training, validation, and test partitions were used for all repeated experiments (see Table 1).

### 4.3. Performance Evaluation Metrics

For comprehensive and objective quantitative assessment of model performance, this paper constructs a multi-level evaluation metric system spanning three dimensions: detection accuracy, computational complexity, and inference latency. In terms of detection accuracy, precision (P) quantifies the fraction of correctly identified positive predictions among all model outputs, reflecting prediction reliability; recall (R) measures how completely all ground-truth targets in the dataset are retrieved, characterizing detection completeness. mAP50 computes the mean of per-class localization accuracy under an IoU criterion of 0.5 and is suited for overall accuracy evaluation under relaxed conditions; mAP50-95 averages mAP across IoU criteria ranging from 0.5 to 0.95 at intervals of 0.05, imposing more rigorous constraints on spatial alignment quality and more finely reflecting bounding box regression quality. In terms of computational complexity, the number of floating-point operations (GFLOPs) and total trainable parameters (Params, M) characterize the per-inference computational burden and model storage overhead, jointly constraining deployment feasibility in resource-constrained scenarios. Inference speed is expressed as frames processed per second (FPS), measured consistently on an NVIDIA RTX 4090 GPU.

Deployment profiling is conducted with a batch size of 1 and an input size of 640×640. Before timing, 100 warm-up iterations are executed, followed by 500 timed images. Preprocessing, network forward inference, and NMS are timed separately, and peak inference memory is recorded using the same software environment as the accuracy experiments. In addition to the RTX 4090 results, the same exported model is profiled on the target edge platform using FP16 inference and identical preprocessing and NMS settings. The latency and memory results are summarized in Table 2.

The deployment profile shows that MarShip-DET introduces a measurable but controlled runtime overhead. On the RTX 4090, total latency increases from 6.64 ms to 7.79 ms per image, while peak memory rises from 812 MB to 1096 MB and throughput decreases from 150.6 FPS to 128.3 FPS. On the Jetson Orin NX 16GB platform with FP16 inference, total latency increases from 28.23 ms to 35.65 ms and peak memory from 1286 MB to 1668 MB, while MarShip-DET maintains 28.1 FPS compared with 35.4 FPS for YOLO11n. These results indicate that the accuracy improvement is accompanied by additional computation and memory consumption, but the model remains capable of near-real-time inference on the evaluated edge platform.

### 4.4. Ablation Study

#### 4.4.1. Component-Level Ablation of the CDPFEM Module

To validate the independent contributions of each key component within CDPFEM, this paper conducts a step-by-step incremental ablation study on the HRSC2016 dataset using YOLO11n as the baseline, systematically evaluating the effects of CDPFEM v1 (the shallow partial convolution and DSCA branch), CDPFEM v2 (the deep image-relative-position-encoded multi-head attention branch), and DSCA on detection accuracy. Experimental results are shown in Table 3.

From the experimental data, the step-by-step introduction of all three components yields continuous performance improvement. Individually introducing CDPFEM v1 improves mAP50-95 by 0.4% over the baseline; further stacking CDPFEM v2 adds 0.7% and 0.3% to mAP50 and mAP50-95, respectively, validating the effectiveness of deep long-range spatial dependency modeling. The subsequent addition of DSCA brings all metrics to optimal values, with mAP50 and mAP50-95 improving by 1.2% and 1.0% over the baseline, respectively, demonstrating the significant contribution of the dual-statistic-driven adaptive channel re-weighting mechanism in enhancing shallow discriminative features.

#### 4.4.2. Ablation Study on Region Decomposition Strategy of EARCFusion

**Figure 5:**
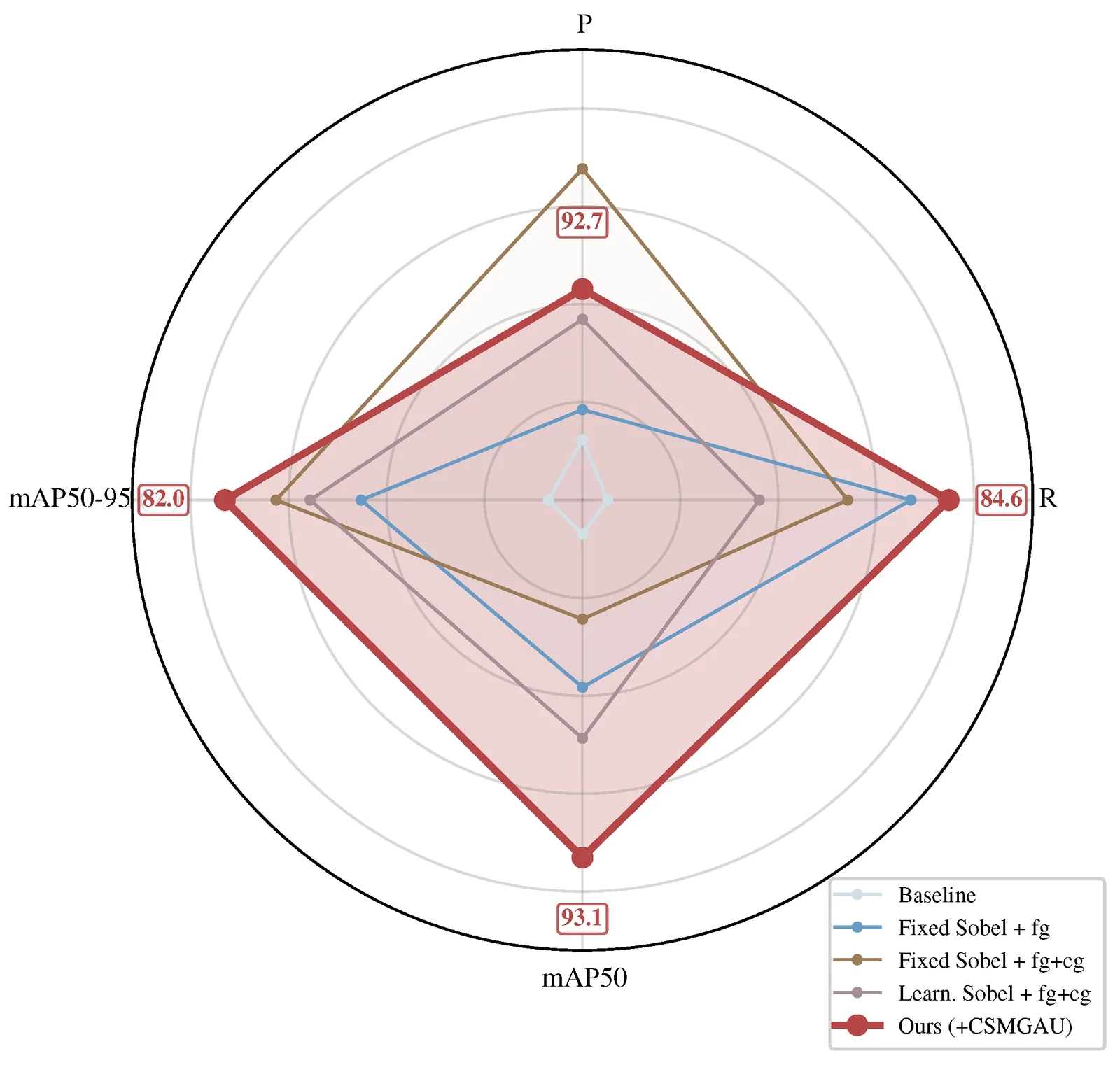
Radar chart comparison of ablation experiments with different region decomposition strategies in the EARCFusion module.

To validate the design rationality of the edge-aware region decomposition mechanism in EARCFusion, this paper systematically evaluates the impact of different combinations of edge detection methods and region partitioning strategies on fusion performance. Experimental results are shown in Table 4 and Figure 5.

Experimental results indicate that each component contributes positively. Introducing the fixed Sobel operator with foreground-only partitioning improves mAP50 by 0.9% over the baseline; further introducing the confusion region partition leads to a marked improvement in recall, indicating that confusion-region guidance effectively reduces the miss-detection rate. Replacing the fixed Sobel with a learnable Sobel further strengthens edge perception capability. With the full incorporation of CSMGAU cross-sample memory-guided attention, the proposed scheme achieves improvements of 1.9% and 1.9% in mAP50 and mAP50-95 over the baseline, validating the effectiveness of the design in which foreground and confusion regions cooperatively drive fine-grained cross-attention correction.

#### 4.4.3. Systematic Module Ablation Study

**Figure 6:**
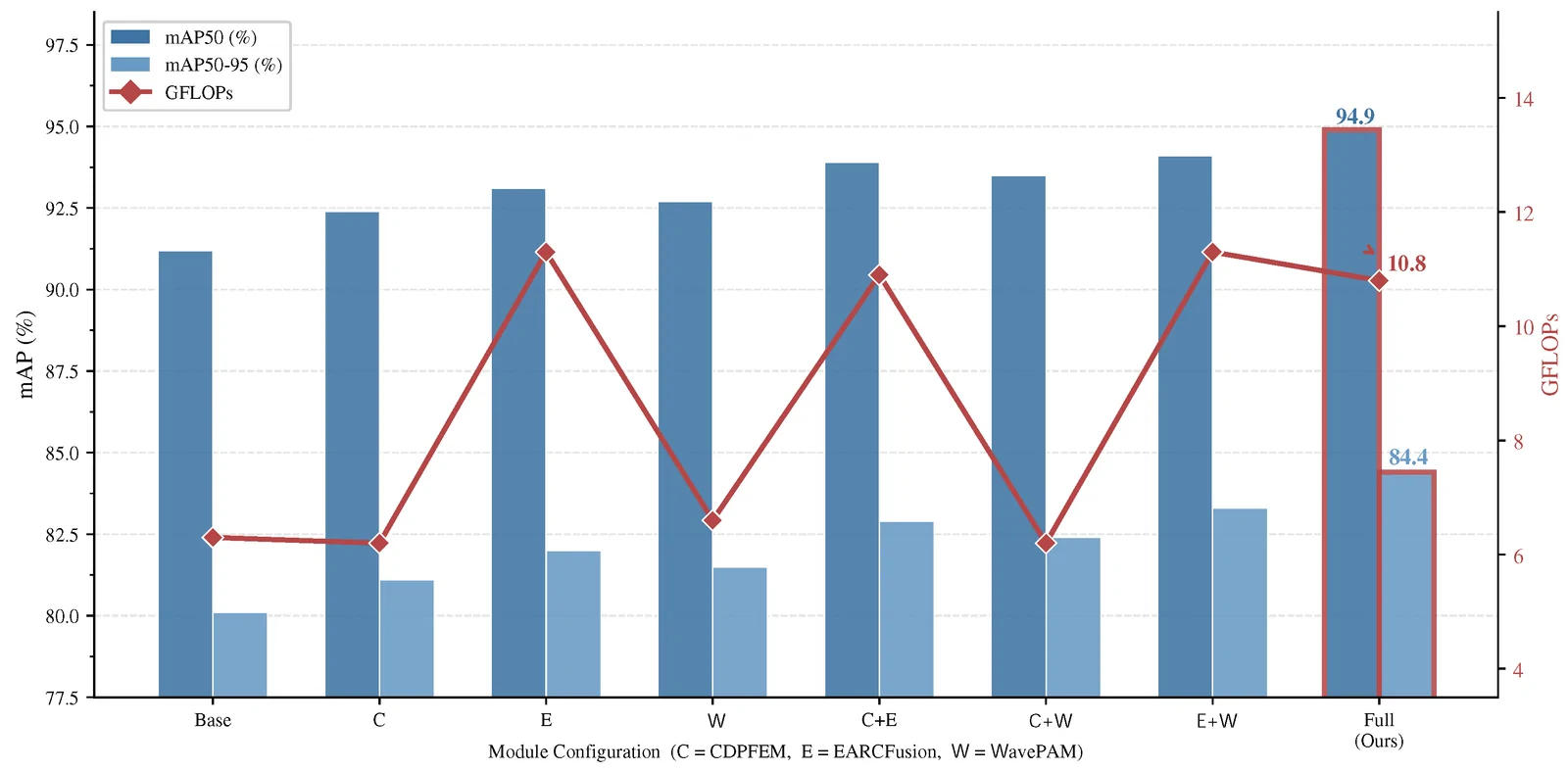
Joint bar chart comparing detection accuracy and computational complexity across the step-by-step module stacking ablation experiments of CDPFEM, EARCFusion, and WavePAM.

To systematically quantify the independent contributions and mutual synergies of the three core improvement modules—CDPFEM, EARCFusion, and WavePAM—on overall model detection performance, this paper designs a comprehensive step-by-step module stacking ablation study on HRSC2016. Using YOLO11n as the reference baseline, detection accuracy metrics and computational complexity are recorded dynamically as modules are added in succession, enabling precise quantification of individual and combined gains. Experimental results are shown in Table 5 and Figure 6.

Among the individual modules, EARCFusion introduces the largest computational overhead, increasing the model by 5.0 GFLOPs and 1.91 M parameters relative to the baseline, whereas CDPFEM slightly reduces both quantities, and WavePAM adds comparatively modest overhead. The full model reaches 10.8 GFLOPs and approximately 4.5 M parameters because the modules interact with the altered backbone and neck rather than contributing strictly additive costs. The deployment profile in Table 6 is used to complement these static complexity indicators with measured latency and memory.

Table 5 reports mean ± standard deviation over three independent random seeds. Across all configurations and accuracy metrics, the standard deviations range from 0.04 to 0.20 percentage points, indicating low run-to-run variation. The complete MarShip-DET model obtains 93.6±0.10% precision, 87.0±0.10% recall, 94.9±0.15% mAP50, and 84.4±0.08% mAP50-95. Relative to the YOLO11n baseline, whose corresponding values are 92.2±0.13%, 81.9±0.10%, 91.2±0.08%, and 80.1±0.17%, the mean mAP50 and mAP50-95 gains are 3.7 and 4.3 percentage points, respectively, while recall increases by 5.1 points. The individual modules also show consistent mean mAP50 gains: CDPFEM reaches 92.4±0.12%, EARCFusion reaches 93.1±0.04%, and WavePAM reaches 92.7±0.11%. Pairwise combinations further increase mAP50 to 93.9±0.16% for CDPFEM+EARCFusion, 93.5±0.09% for CDPFEM+WavePAM, and 94.1±0.13% for EARCFusion+WavePAM. The complete model therefore achieves the highest mean mAP50 and mAP50-95 while maintaining small standard deviations, supporting the stability of the observed improvements across the evaluated random seeds.

#### 4.4.4. Heatmap Visualization

**Figure 7:**
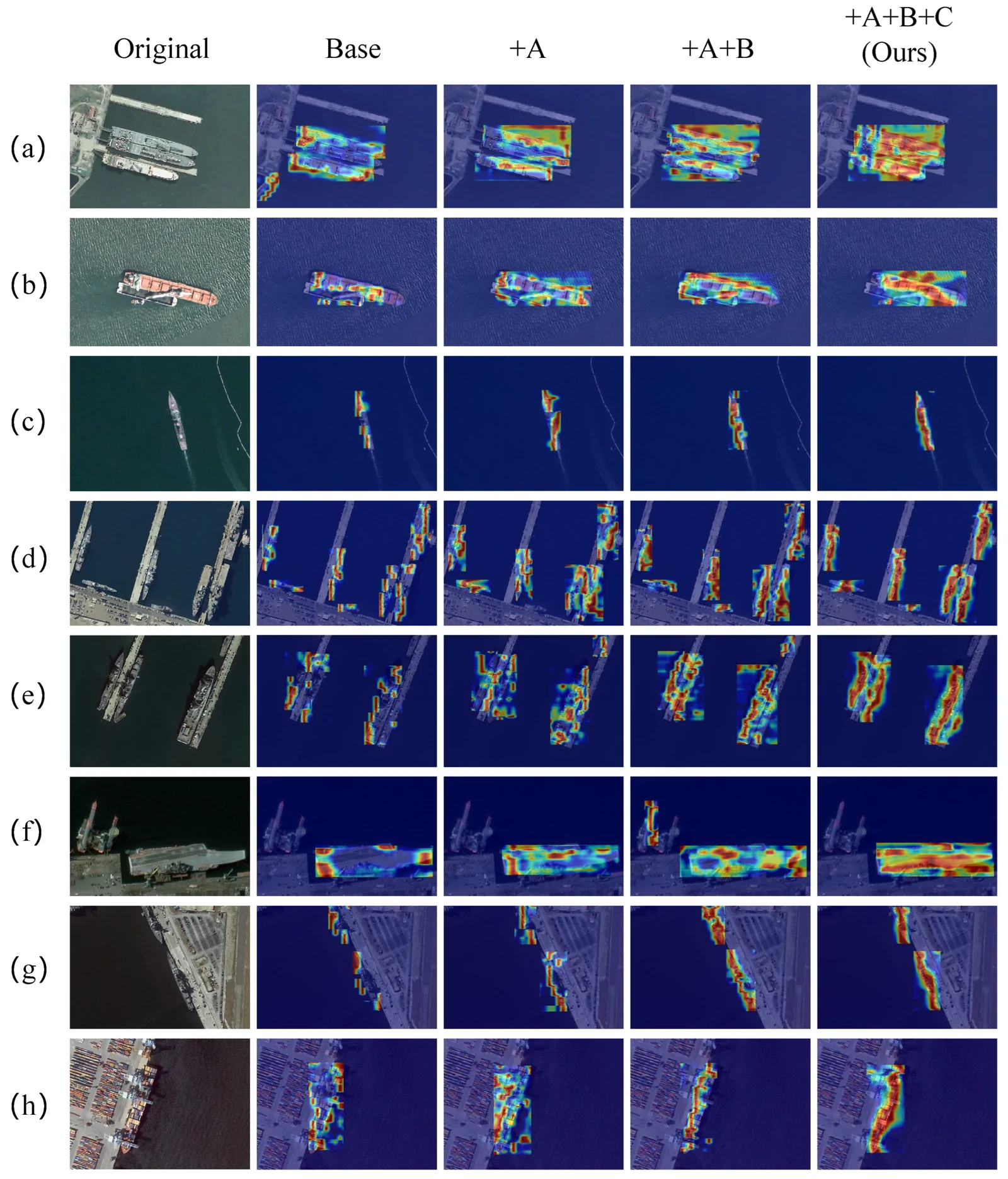
Grad-CAM heatmap visualization comparison between the baseline model and various ablation configurations. Rows (**a**–**h**) correspond to eight randomly selected samples from the dataset.

To further validate the effectiveness of the three improvement modules from a feature response perspective, this paper employs gradient-weighted class activation mapping (Grad-CAM) to visualize feature activation heatmaps under each ablation configuration. Eight representative scenes from the HRSC2016 test set are selected—covering dense harbor anchoring, single-ship open water, distant small targets, and strong background interference—to systematically present the evolution of feature response patterns. Results are shown in Figure 7.

The Grad-CAM visualizations show that the baseline responses are relatively diffuse in several dense-port and coastal-background examples, whereas the responses after introducing CDPFEM and EARCFusion become more concentrated around ship regions. With the complete configuration, the displayed examples show stronger activation near ship bodies and clearer separation from surrounding clutter. These qualitative observations are consistent with the quantitative improvements reported in the ablation study, but Grad-CAM is an interpretive visualization and does not by itself establish localization accuracy or robustness across all scenes.

### 4.5. Comparison Experiments

#### 4.5.1. Lateral Comparison of Backbone Feature Extraction Modules

This paper systematically compares CDPFEM with representative improved variants, including C3k2-DEGConv [25], C3k2-MSInit [26], C3k2-PFG [26], and C3k2-SMB [27], replacing only the feature extraction module while keeping the remaining backbone structure unchanged to ensure fair comparison. Experimental results are shown in Table 7.

Relative to C3k2-MSInit, which has the smallest parameter count, CDPFEM improves mAP50 and mAP50-95 by 2.0% and 1.9%, respectively; relative to C3k2-DEGConv incorporating deformable convolution, CDPFEM reduces the parameter count by 0.38 M and GFLOPs by 0.6 while improving mAP50 and mAP50-95 by 0.6% each; relative to the closest-performing C3k2-PFG, CDPFEM achieves a 0.3% mAP50 improvement at lower computational cost. Under the unified comparison setting in Table 7, CDPFEM records the highest mAP50 and mAP50-95 while retaining relatively low computational cost, supporting the effectiveness of the channel-decoupled and depth-adaptive attention design.

#### 4.5.2. Lateral Comparison of Neck Feature Fusion Modules

To validate EARCFusion in multi-scale feature fusion, this paper compares it with representative fusion modules including LCA [28], FAAFusion [29], HFFE [30], and GDSAFusion [31] under identical experimental conditions. Experimental results are shown in Table 8.

FAAFusion and GDSAFusion use fewer computational resources than EARCFusion but record lower mAP50 values in Table 8. Compared with HFFE, EARCFusion uses 1.1 fewer GFLOPs and records a 1.2-percentage-point higher mAP50 under the reported setting. Within this module-level comparison, EARCFusion obtains the highest recorded mAP50 and mAP50-95, suggesting that edge-aware region decomposition and region-conditioned cross-attention can improve fusion quality, although the additional parameter cost should be considered.

#### 4.5.3. Comprehensive Performance Comparison with Mainstream Detection Algorithms

For a comprehensive evaluation of MarShip-DET, this paper conducts a lateral comparison on HRSC2016 with multiple mainstream detection frameworks spanning DETR-series and YOLO-series methods, covering DETR-family methods including Deformable-DETR [32], DINO [33], and D-FINE-N [34], as well as one-stage detectors from YOLOv5 through YOLOv13. Experimental results are shown in Table 9.

All detector comparisons in Table 9 are retrained using the same official HRSC2016 split, 640×640 input resolution, 200 training epochs, augmentation policy, hardware/software environment, and evaluation code. No literature-reported accuracy value is directly copied into Table 9.

To ensure a fair comparison, all detectors in Table 9 were trained and evaluated under the same experimental protocol: the official HRSC2016 training/validation/test split, 640×640 input resolution, 200 training epochs, the same data-augmentation policy, identical hardware and software environments, and the same evaluation code. The implementations corresponding to the cited methods were retrained under this unified setting, and the accuracy values in Table 9 were obtained from these controlled experiments rather than copied from the original publications. Under this protocol, MarShip-DET records 94.9% mAP50 and 84.4% mAP50-95, outperforming the YOLO11n baseline by 3.7 and 4.3 percentage points, respectively, with a 5.1-point recall increase. Compared with DINO, D-FINE-N, and YOLOv13n, MarShip-DET also records a higher mAP50 and mAP50-95 under the same evaluation setting. The accuracy improvement is accompanied by increased computational cost: GFLOPs rise from 6.3 to 10.8 and parameters from 2.62 M to 4.51 M relative to YOLO11n, while FPS decreases from 150.6 to 128.3. Thus, the results demonstrate a clear accuracy–efficiency trade-off rather than a cost-free improvement.

### 4.6. Generalization Experiments

For the cross-domain experiments, zero-shot transfer is defined as direct inference on the target SAR datasets using weights trained exclusively on HRSC2016, without target-domain training, fine-tuning, domain adaptation, hyperparameter tuning, threshold selection, or model screening. Single-channel SAR images are replicated to three channels, followed by the same letterbox resizing to 640×640 and the same numerical scaling used by the YOLO11 evaluation pipeline. HRSID and SSDD annotations are converted to the single ship class in normalized YOLO horizontal-box format. When an original annotation is polygonal or oriented, its minimum enclosing axis-aligned rectangle is used to remain consistent with the horizontal detection head. All target-domain models use the same confidence threshold of 0.001, NMS IoU threshold of 0.7, and maximum detection count of 300. No HRSID or SSDD image or label is used to select the source-domain checkpoint (see Table 10).

The cross-domain evaluation uses the same zero-shot protocol for YOLO11n, D-FINE-N, YOLOv13n, and MarShip-DET. On HRSID, MarShip-DET achieves the highest recall (85.2%), mAP50 (91.9%), and mAP50-95 (69.3%) among the four models. Relative to D-FINE-N, it improves recall, mAP50, and mAP50-95 by 1.7, 0.7, and 0.6 percentage points, respectively; relative to YOLOv13n, the corresponding gains are 1.2, 0.4, and 1.2 points. On SSDD, MarShip-DET obtains the highest values across all four accuracy metrics, reaching 96.7% precision, 93.9% recall, 97.8% mAP50, and 72.4% mAP50-95. Compared with D-FINE-N, these values correspond to gains of 0.9, 0.3, 0.7, and 0.2 points, while the gains over YOLOv13n are 0.6, 1.0, 0.4, and 0.4 points, respectively. The consistent improvements across both SAR datasets indicate that MarShip-DET retains useful cross-domain transferability under the evaluated protocol, although the higher 10.8 GFLOPs should be considered when interpreting the accuracy–efficiency trade-off.

### 4.7. Scale-Stratified and Scenario-Oriented Diagnostic Analysis

To determine whether the performance gain is concentrated at a particular vessel scale, the HRSC2016 test set was additionally evaluated with COCO-style area bins after mapping all horizontal boxes to the common 640×640 evaluation coordinate system: small objects have an area of <32^2^ pixels, medium objects have an area from $32^{2}$ to $96^{2}$ pixels, and large objects have an area of >96^2^ pixels. Table 11 reports the corresponding AP values.

**Figure 8:**
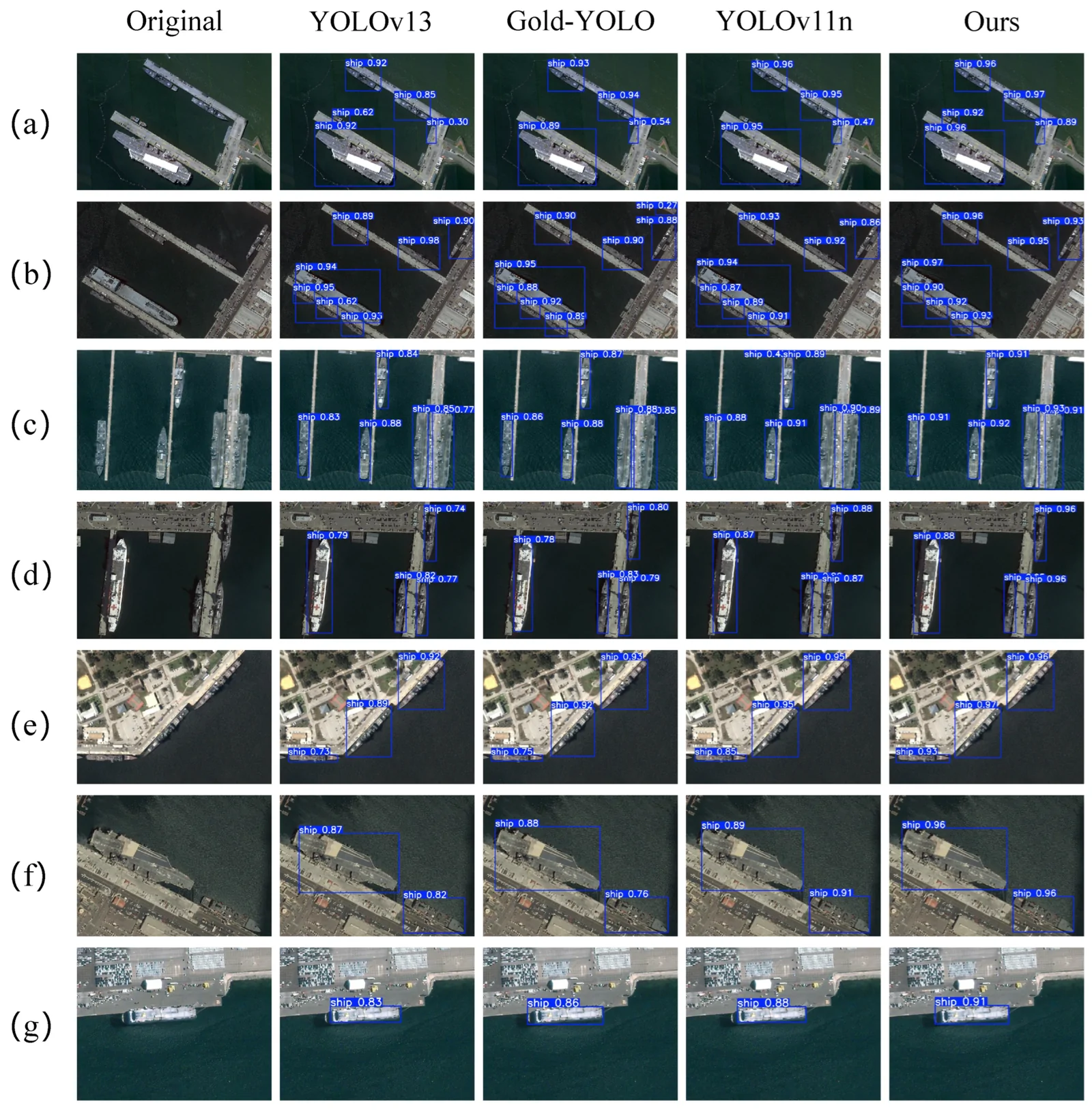
Per-scene detection result visualization of MarShip-DET, YOLOv13n, Gold-YOLO-n, and YOLO11n across seven representative challenging scenarios from the HRSC2016 test set. Rows (**a**–**g**) correspond to seven randomly selected samples from the dataset.

The scale-stratified results show that MarShip-DET improves AP_*s*_ from 68.7% to 75.4%, a gain of 6.7 percentage points. AP_*m*_ increases from 81.3% to 85.0% (+3.7 points), while AP_*l*_ rises from 85.6% to 87.2% (+1.6 points). The largest gain occurs for small vessels, indicating that the proposed multi-scale and frequency-aware mechanisms are particularly effective for targets occupying a limited image area; the simultaneous positive gains for medium and large vessels show that the improvement is not confined to small objects. The dense-port, near-shore, distant-small-target, and partially occluded cases in Figure 8 further complement the scale-stratified metrics with scene-oriented evidence of reduced missed detections and localization offsets.

### 4.8. Visualization

To intuitively illustrate the practical detection performance differences of MarShip-DET relative to mainstream detection algorithms, this paper selects seven representative challenging detection scenarios from the HRSC2016 test set, covering dense warship anchoring, distant small targets, strong perspective distortion, near-shore complex backgrounds, and partial target occlusion, and presents per-scene visualization comparisons against YOLOv13n, Gold-YOLO-n, and YOLO11n. Results are shown in Figure 8.

The qualitative comparisons in Figure 8 cover dense anchoring, distant small targets, strong perspective variation, near-shore background interference, and partial occlusion. In the displayed examples, MarShip-DET produces fewer visible misses or box offsets than the compared outputs in several challenging scenes and maintains relatively high confidence on distant targets. These examples provide scene-level diagnostic evidence that complements the aggregate mAP metrics, but they are not a substitute for scale-stratified AP, precision–recall curves, localization-error statistics, or a systematic failure-case study.

## 5. Conclusions

MarShip-DET modifies YOLO11n through three coordinated components for channel-decoupled feature extraction, edge-aware region-context fusion, and frequency-guided prototype attention. Under the reported HRSC2016 setting, the complete model records 94.9% mAP50 and 84.4% mAP50-95, compared with 91.2% and 80.1% for the YOLO11n baseline. Repeated-seed experiments show low variation, with standard deviations of 0.15 and 0.08 percentage points for the full model’s mAP50 and mAP50-95, respectively. Under the unified zero-shot protocol, MarShip-DET also records the highest mAP50 and mAP50-95 among YOLO11n, D-FINE-N, YOLOv13n, and MarShip-DET on both HRSID and SSDD. These accuracy gains are accompanied by higher computational cost: GFLOPs increase from 6.3 to 10.8, parameters from 2.62 M to approximately 4.5 M, and RTX 4090 throughput decreases from 150.6 FPS to 128.3 FPS; on Jetson Orin NX, MarShip-DET maintains 28.1 FPS with a 35.65 ms total latency.

Several limitations remain. The model is trained on a single optical dataset, and the cross-domain study is limited to two SAR datasets even though multiple comparison detectors are now included. Broader validation across additional optical, SAR, and multimodal domains is therefore still required before claiming universal cross-sensor robustness. The proposed modules also increase latency and memory consumption relative to YOLO11n, as shown by the deployment profile, so further compression remains important for resource-constrained platforms. Future work will extend the study to oriented-box detection, multi-polarization SAR, nighttime optical imagery, multimodal fusion, and structured model compression.

## Figures and Tables

**Table 1:** Key implementation settings of the three proposed modules.

Module	Implementation Setting
CDPFEM	$n_{\mathrm{div}}=4$; active local subset uses a 3×3 partial convolution; shallow global subset uses mean–standard deviation DSCA; deep global subset uses image-relative-position multi-head attention.
EARCFusion	Adjacent features are aligned by a 1×1 convolution and fused by a 3×3 convolution; Sobel kernels are learnable; the region probability map is centered by the fixed 0.5 offset; *S* denotes the number of CSMGAU memory slots, as defined in Equation (5).
WavePAM	One-level fixed Haar decomposition is used; high-frequency subbands are summed before LGDCWU weighting; *M* denotes the number of learnable prototype vectors in the prototype branch; the two attention branches are merged by residual addition.

**Table 2:** Deployment-oriented latency and memory profile at 640×640 input resolution and a batch size of 1.

Platform	Model	Preprocess/ms	Forward/ms	NMS/ms	Total/ms	Peak Memory/MB	FPS
RTX 4090	YOLO11n	0.72	5.38	0.54	6.64	812	150.6
RTX 4090	MarShip-DET	0.74	6.42	0.63	7.79	1096	128.3
Jetson Orin NX 16GB (FP16)	YOLO11n	1.86	24.73	1.64	28.23	1286	35.4
Jetson Orin NX 16GB (FP16)	MarShip-DET	1.92	31.84	1.89	35.65	1668	28.1

**Table 3:** Step-by-step component ablation results of the CDPFEM module.

Model	CDPFEM v1	CDPFEM v2	DSCA	P	R	mAP50	mAP50-95
1. base				92.2	81.9	91.2	80.1
2	✓			92.4	82.5	91.1	80.5
3	✓	✓		92.6	82.3	91.8	80.8
4. ours	✓	✓	✓	93.2	82.9	92.4	81.1

**Table 4:** Ablation results of region decomposition strategies in the EARCFusion module.

Exp.	Edge Detection	Region Strategy	P	R	mAP50	mAP50-95
1. base	None	No decomposition	92.2	81.9	91.2	80.1
2	Fixed Sobel	Foreground only (fg)	92.3	84.3	92.1	81.2
3	Fixed Sobel	Foreground + confusion (fg + cg)	93.1	83.8	91.7	81.7
4	Learnable Sobel	Foreground + confusion (fg + cg)	92.6	83.1	92.4	81.5
5. ours	Learnable Sobel	fg + cg + CSMGAU	92.7	84.6	93.1	82.0

**Table 5:** Systematic module ablation results on HRSC2016. Accuracy metrics are reported as mean ± standard deviation over three independent runs.

Model	CDPFEM	EARCFusion	WavePAM	GFLOPs	Params/M	P	R	mAP50	mAP50-95
1. base				6.3	2.62	92.2 ± 0.13	81.9 ± 0.10	91.2 ± 0.08	80.1 ± 0.17
2	✓			6.2	2.46	93.2 ± 0.07	82.9 ± 0.10	92.4 ± 0.12	81.1 ± 0.09
3		✓		11.3	4.53	92.7 ± 0.15	84.6 ± 0.20	93.1 ± 0.04	82.0 ± 0.14
4			✓	6.6	2.79	92.9 ± 0.09	83.4 ± 0.20	92.7 ± 0.11	81.5 ± 0.07
5	✓	✓		10.9	4.31	93.4 ± 0.18	85.4 ± 0.10	93.9 ± 0.16	82.9 ± 0.12
6	✓		✓	6.2	2.63	93.6 ± 0.06	84.1 ± 0.20	93.5 ± 0.09	82.4 ± 0.15
7		✓	✓	11.3	4.71	92.7 ± 0.12	85.8 ± 0.10	94.1 ± 0.13	83.3 ± 0.10
8. ours	✓	✓	✓	10.8	4.5	93.6 ± 0.10	87.0 ± 0.10	94.9 ± 0.15	84.4 ± 0.08

**Table 6:** Component-wise changes in computational complexity relative to YOLO11n.

Configuration	ΔGFLOPs	ΔParams/M	ΔmAP50	ΔmAP50-95
CDPFEM only	−0.1	−0.16	+1.2	+1.0
EARCFusion only	+5.0	+1.91	+1.9	+1.9
WavePAM only	+0.3	+0.17	+1.5	+1.4
Full MarShip-DET	+4.5	+1.88	+3.7	+4.3

**Table 7:** Lateral comparison of backbone feature extraction modules.

Model	GFLOPs	Params/M	P (%)	R (%)	mAP50 (%)	mAP50-95 (%)
C3k2 (base)	6.3	2.62	92.2	81.9	91.2	80.1
C3k2-DEGConv [25]	6.8	2.84	92.6	82.1	91.8	80.5
C3k2-MSInit [26]	5.7	2.13	91.5	80.8	90.4	79.2
C3k2-PFG [26]	6.5	2.71	92.9	82.3	92.1	80.8
C3k2-SMB [27]	5.9	2.28	92.5	82.1	91.7	80.4
CDPFEM (ours)	6.2	2.46	93.2	82.9	92.4	81.1

**Table 8:** Lateral comparison of neck feature fusion modules.

Model	GFLOPs	Params/M	P	R	mAP50	mAP50-95
Concat (base)	6.3	2.62	92.2	81.9	91.2	80.1
LCA [28]	7.1	3.12	91.8	82.1	91.6	80.4
FAAFusion [29]	5.8	2.15	91.5	81.2	90.7	79.5
HFFE [30]	12.4	4.15	92.6	82.8	91.9	80.8
GDSAFusion [31]	6.1	2.51	92.1	82.4	91.5	80.5
EARCFusion (ours)	11.3	4.53	92.7	84.6	93.1	82.0

**Table 9:** Comprehensive performance comparison with mainstream detection algorithms on HRSC2016.

Model	GFLOPs	Params/M	P	R	mAP50	mAP50-95	FPS
DETR-Based Object Detectors							
Deformable-DETR [32]	173.2	40.12	88.4	76.5	87.2	65.4	19.3
DINO [33]	166.4	47.35	91.2	81.4	91.5	78.2	24.7
D-FINE-N [34]	7.1	4.05	92.1	83.1	92.4	80.5	150.8
One-Stage Object Detectors							
YOLOv5n [35]	4.5	1.86	86.4	75.2	84.9	68.3	185.4
YOLOv5s [35]	16.5	7.23	88.2	78.6	87.8	71.2	135.6
YOLOv8n [36]	8.1	3.16	89.2	79.5	89.1	75.4	162.1
YOLOv9t [37]	7.6	2.05	88.7	78.4	88.5	74.2	170.9
YOLOv10n [38]	6.4	2.27	90.8	80.6	90.5	77.3	158.3
YOLOv12n [39]	6.5	2.58	91.9	81.3	91.6	79.8	142.5
YOLOv13n [40]	7.2	2.84	92.5	82.7	92.2	81.2	135.7
PP-YOLOE-s [41]	17.4	7.93	89.5	78.2	88.7	73.5	120.2
DAMO-YOLO-T [42]	8.4	3.21	89.8	80.1	89.6	76.2	152.8
Gold-YOLO-n [43]	10.6	3.96	91.2	81.5	91.1	78.8	138.4
YOLO11n (base) [18]	6.3	2.62	92.2	81.9	91.2	80.1	150.6
MarShip-DET (ours)	10.8	4.51	93.6	87.0	94.9	84.4	128.3

**Table 10:** Zero-shot generalization results on HRSID and SSDD under the same target-domain preprocessing and evaluation protocol.

Dataset	Model	GFLOPs	P	R	mAP50	mAP50-95
HRSID	YOLO11n (base) [18]	6.3	92.4	81.9	90.7	67.4
	D-FINE-N [34]	7.1	92.6	83.5	91.2	68.7
	YOLOv13n [40]	7.2	91.8	84.0	91.5	68.1
	MarShip-DET (ours)	10.8	91.6	85.2	91.9	69.3
SSDD	YOLO11n (base) [18]	6.3	94.9	93.4	96.7	72.1
	D-FINE-N [34]	7.1	95.8	93.6	97.1	72.2
	YOLOv13n [40]	7.2	96.1	92.9	97.4	72.0
	MarShip-DET (ours)	10.8	96.7	93.9	97.8	72.4

**Table 11:** Scale-stratified AP comparison on HRSC2016.

Model	AP_*s*_	AP_*m*_	AP_*l*_
YOLO11n (base)	68.7	81.3	85.6
MarShip-DET (ours)	75.4	85.0	87.2

## Data Availability

The HRSC2016 dataset is publicly available at https://dx.doi.org/10.21227/km4k-0385. The HRSID dataset and SSDD dataset are available upon reasonable request from the respective original authors.
